# Molecular variants of multiple genes were revealed by whole-exome sequencing in PCOS patients with diabetes

**DOI:** 10.3389/fgene.2025.1541946

**Published:** 2025-05-23

**Authors:** Chenglin Wang

**Affiliations:** Department of Endocrinology, Shanxi Provincial People’s Hospital, Taiyuan, China

**Keywords:** polycystic ovary syndrome, diabetes, whole-exome sequencing, bioinformatics analysis, AR gene, insulin resistance

## Abstract

**Objective:**

To screen for possible pathogenic mutations in polycystic ovary syndrome (PCOS) patients with diabetes and preliminarily explore the relationship between genotype and phenotype to offer a research basis for PCOS pathogenesis with diabetes.

**Methods:**

Four patients with PCOS and diabetes were recruited and their demographic and clinical data were collected. Genomic DNA was extracted from peripheral blood leukocytes of the study subjects. High-throughput whole-exome sequencing was conducted to identify candidate genes that could play a pathogenic role in PCOS with diabetes in Aiji Taikang. The sequencing data obtained were evaluated using a variety of bioinformatics tools. Verification of candidate sites was done by Sanger sequencing.

**Results:**

Based on whole-exome sequencing, six mutations residing in three genes were detected in these four patients: (1) *MUC4* located at Chr 3q29, (2) FSHD region gene 1 (*FRG1*)gene located at Chr 4q35.2, and (3) androgen receptor (*AR*) located at Chr Xq11-q12 were detected in these four patients (every patients had the 6 mutations). Of the six genetic mutations, an insertion/deletion (indel) mutation was found in the mucin 4 (*MUC4*) gene [MUC4:NM_018406.6:2/25:c.7701_7702insTCAGTATCCACAGGTCATGCCACCCCTCTTCATGTCACCGACACTTCC:p.(Ser2567_Ala2568insSerValSerThrGlyHisAlaThrProLeuHisValThrAspThrSer)], and an indel mutation in the *AR* gene (*AR*:NM_000044:exon1:c.173_174insGCAGCA:p. Q58delinsQQQ), while the other four were missense single-nucleotide polymorphisms (SNPs) located in *FRG1* of uncertain significance (*FRG1*:NM_004477:exon8:c.T692C:p. L231P, *FRG1*:NM_004477:exon8:c.C728T:p.T243M, *FRG1*:NM_004477:exon8:c.C733A:p.L245M, FRG1:NM_004477:exon8:c.T734G:p.L245R). A Mucin 4 (*MUC4*) gene indel mutation was detected at the same site in four patients, which could be associated with endometriosis-related infertility. The *AR* gene indel mutation, *AR*:NM_000044:exon1:c.173_174insGCAGCA: p. Q58delinsQQQ was detected simultaneously in four patients.

**Conclusion:**

Whole exome sequencing can quickly identify candidate genes for genes. Gaining an in-depth understanding of the AR mutations underlying PCOS with diabetes will deepen our understanding of the endocrine factors involved in the disease etiology, and provide potential targets for treatment.

## Introduction

Polycystic ovary syndrome (PCOS) is a highly prevalent, heterogeneous disease in women of childbearing age. It is characterized by anovulation, irregular menstruation, amenorrhea, hirsutism, and infertility. Additionally, diverse metabolic symptoms have been reported, including insulin resistance (IR) diabetes, obesity, extensive coronary artery disease, hypertension, endometrial hyperplasia, ovarian cancer, and breast cancer (Al-Saleh, 2022; Chen et al., 2021; Smith et al., 2021; Falcetta et al., 2021). Currently, the Rotterdam consensus criteria have been used for PCOS diagnosis, and at least two of the following items must be met: hyperandrogenemia, oligomenorrhea or amenorrhea, and polycystic ovarian changes (unilateral or bilateral ovaries 2–9 mm, number of follicles ≥ 12, or ovarian volume ≥10 mL, ovarian volume = 0.5 × length × width × thickness) (Lauritsen et al., 2014). Treatment options and interventions include lifestyle adjustments, hyperandrogenemia, insulin resistance, recommended metformin use, and ovulation induction therapy in patients with fertility requirements.

Diverse risk factors are associated with PCOS in adults, including obesity, low exercise, high serum glucose levels, insulin resistance, type 1 or 2 diabetes, gestational diabetes, and mental health disorders. In recent years, research on PCOS pathogenesis has focused predominantly on genome-wide association studies, androgen biosynthesis and metabolism, and oxidative stress cycle markers. However, PCOS is a common female endocrine disorder that affects women of reproductive age and is often associated with metabolic diseases, including diabetes. Despite rigorous efforts, the genetic variants underlying PCOS associated with diabetes remain largely unknown.

Polycystic ovary syndrome (PCOS) is a multifactorial and polygenic disease involving a spectrum of environmental and lifestyle factors, and multiple genetic mutations. For example, genes, such as androgen receptor (*AR)* and sex hormone-binding globulin(*SHBG*), are involved in steroid hormone changes (Khan et al., 2019; Muccee et al., 2022; Samant et al., 2025; Villuendas et al., 2003). Several researchers have reported *AR* mutations in PCOS patients (Hickey et al., 2002; Wang et al., 2015; Shah et al., 2008; Schüring et al., 2012). Several studies have demonstrated that PCOS incidence is familial and inherited predominantly in an autosomal dominant manner. PCOS is a complex polygenic disease with an X-linked dominant inheritance or autosomal dominant inheritance pattern that does not conform to the Mendelian inheritance law. Most studies suggest that PCOS is a syndrome of endocrine and metabolic abnormalities caused by heredity, environment, and lifestyle, wherein genetic factors play an important role.

Exome sequencing is a high-throughput sequencing method that can simultaneously detect all exon mutations in the human genome at one time, which is of significant value for exploring disease-related genes (Azad et al., 2024; Dauda et al., 2022; Aqeel et al., 2022). Several studies have identified disease-modifying genes using exonic approaches. Several studies on PCOS have been conducted using exome sequencing. Herein, PCOS-associated genes were investigated using exome sequencing. The aim of this study was to identify potential pathogenic mutations in patients with polycystic ovary syndrome (PCOS) and diabetes and to investigate the genotype-phenotype relationship, thereby offering a research foundation for understanding PCOS pathogenesis associated with diabetes.

## Materials and methods

In this case-control study, four women were recruited from Shanxi Provincial People’s Hospital. Among these subjects who were all Han Chinese women, were diagnosed as PCOS according to the Rotterdam diagnostic criteria. None of the subjects had any other diseases, and none had undergone chemotherapy or radiotherapy. All were unrelated individuals of reproductive age who had not received hormonal therapy for at least 3 months prior to the study.

### The clinical examinations conducted on the patients

Patients 1 and 2 were diagnosed with diabetes using a 75 g Oral Glucose Tolerance Test (OGTT) at 4 weeks postpartum, fasting (7.5 mM), and 2-h blood glucose 11.5 mmol/L. Patient 2, her blood sugar was elevated, her fasting blood sugar was 8.5 mmol/L, and her 2-h postprandial blood sugar was 15.6 mmol/L. The third patient had normal blood sugar. Patient 4 measured 12.5 mmol/L of blood glucose 2 h post meals,sex hormone test demonstrated that her testosterone level was elevated.

Based on insulin release, all four patients had insulin resistance. All four patients had normal prolactin.

These four patients had normal FSH, LH, and progesterone. The patient 1, 2, and 3 had normal androgen and the fourth patient had elevated androgen.

### Whole-exome sequencing and bioinformatics analysis

#### DNA extraction

A 5 mL venous blood sample was collected from each patient. Heparin was used as the anticoagulant. Human peripheral blood leukocyte genomic DNA was extracted using the OMEGA SE Blood DNA Kit and sent to Aiji Taikang for whole-exome sequencing (Heald et al., 2020).

#### High-throughput whole-sequencing sequencing

Genomic DNA samples from the four patients were sent to Aiji Taikang for whole sequencing using the Illumina NovaSeq 6000 sequencing platform and paired-end sequencing.

#### Bioinformatics analysis of exome sequencing

The sequencing quality of the raw sequence reads was evaluated by FastQC (De Sena Brandine and Smith, 2019), and low-quality reads were removed by Trimmomatic to obtain high-quality sequences, which were aligned with the human reference genome (hg19) by Burrows-Wheeler Aligner (BWA) (Li and Durbin, 2009). Duplicates were marked by samblaster (Faust and Hall, 2014) by comparing read sequences with control sequences. GATK HaplotypeCaller (McKenna et al., 2010) was used for mutation analysis, and the National Center for Biotechnology Information (NCBI) dbSNP and ClinVar, 1000 Genomes database, dbNSFP (Liu et al., 2016), and other databases were used to annotate the population frequencies of the variant sites.

#### Analysis of mutations and candidate genes

Populations with mutation frequencies greater than 1% and mutations with no biological significance (intron regions, synonymous mutations, and other mutations that did not affect protein function) were filtered out. The intersection of the mutations in the four patients was evaluated.

#### Verification of candidate sites

The *AR* gene mutation site was verified by first-generation sequencing using primers with the sequences 5′ AAG​ACC​TAC​GAG​GAG​CTT​TCC 3′ and 5′ TTG​GGG​AGA​ACC​ATC​CTC​ACC 3′. PCR was conducted using a Bio-Rad T100 for amplification. PCR system: 1 µL DNA template, 1 µL each of upstream and downstream primers, 0.5 µL Taq enzyme (TAKARA), 5 µL dNTP, 4 µL buffer, and the remaining volume adjusted to 50 μL with double-distilled water. The reaction conditions were as follows: pre-denaturation at 94°C for 3 min; denaturation at 94°C for 30 s; annealing temperature at 56°C for 30 s; extension at 72°C for 45 s; and extension at 72°C for 5 min post 30 cycles. The resulting PCR products were stored at 4°C. Post the reaction, the cells were electrophoresed on a 1% agarose gel, stained with ethidium bromide, and the bands were observed under UV light. The PCR products were sent to Shanghai Shenggong Biological Company for sequencing. The obtained sequence was aligned with each exon sequence offered by GenBank and further searches were conducted to determine whether these mutations were present in the NCBI dbSNP or Human Gene Mutation Database (HGMD).

## Results

All four patients were Asian. All patients exhibited insulin resistance, hyperandrogenemia, and normal prolactin levels.

### History of present illness

Patient one was a 32 years old. In 2003, the patient experienced menstrual disorders for no obvious reason. In February 2018, she became pregnant post ovulation induction. Her blood glucose levels increased during the pregnancy. The patient was diagnosed with “gestational diabetes.” Type 2 diabetes was diagnosed a 75 g Oral Glucose Tolerance Test (OGTT) at 4 weeks postpartum, fasting (7.5 mM), and 2-h blood glucose 11.5 mmol/L according to the 2020 Chinese Diabetes Prevention and Treatment Guidelines. The sequencing data were obtained during pregnancy.

Patient 2 is female, 31 years old. In June 2019, she visited our hospital due to amenorrhea for 3 months. Simultaneously, her blood sugar level was elevated; her fasting blood sugar was 8.5 mmol/L, and her 2-h postprandial blood sugar was 15.6 mmol/L.

Patient 3, female 21 years old. She visited a doctor for menstrual disorders with symptoms of dry mouth, polydipsia, polyuria, and weight loss.

Patient 4 is female, 32 years old. She visited the dermatology department of our hospital with acne. Sex hormone tests revealed elevated testosterone levels. The patient was transferred to our department. The patient denied menstrual disorders and claimed to have normal menstrual flow. She had been experiencing dry mouth recently and measured 12.5 mmol/L blood glucose 2 h post meals at home.

Patient 1 was 168 cm tall, weighed 85 kg, had a 124/70 mmHg blood pressure, and body mass index (BMI) of 30 kg/m^2^. She was obese, hirsute, and had visible acne on her face, acanthosis nigricans and no edema in the lower limbs. Physical examination of the remaining three patients demonstrated no obvious abnormalities. All four patients had a family history of polycystic ovaries and diabetes. General clinical data included routine blood tests, liver function, renal function, glycosylated hemoglobin, cortisol rhythm, gynecological color Doppler ultrasonography, thyroid function, androgen and prolactin levels, adrenal CT), and electrocardiography. This study was approved by the Research Ethics Commission of the Shanxi Provincial People’s Hospital (201992).

### Laboratory information

Patient 1: 8 points adrenocorticotropic hormone (ACTH) 38.66 (6.0–40) pg/mL, fasting blood glucose 11.59 mmol/L, glycosylated hemoglobin 10.5%.

Normal thyroid function, 8 points of cortisol 159.7 ng/mL (72.6–233.8), 4 points of 98.87 ng/mL, 0 points of cortisol 27.33 ng/mL, 24-h urine free cortisol 305.24 µg/24 h, follicle-stimulating hormone (FSH) 6.711 mIU/mL, luteinizing hormone (LH) 8.088 mIU/mL, testosterone 0.342 (0–1) ng/mL.

Patient 1 was diagnosed with diabetes based on fasting blood glucose and A1C. Patient 1’s cortisol rhythm and ACTH levels were normal, except for hypercortisolism. Patient 1 had normal levels of sex hormones.

Patient 2: 8 points of cortisol 167.43 ng/mL (72.6–233.8) 4 points of 86.54, 0 points of cortisol 30.00, 24-h urine free cortisol 220.00 µg/24 h, glycosylated hemoglobin 8.5%.

Patient 2’s cortisol rhythm and ACTH levels were normal, except for hypercortisolism. The second patient had diabetes mellitus.

Patient 3: 8 points cortisol 233.0 ng/mL (72.6–233.8) 4 points 142.5 ng/mL, 0 points 60.00 ng/mL, 24 h urinary free cortisol 125.554 µg/24 h, 8 points ACTH 37.17 (6.0–40) pg/mL, glycosylated hemoglobin 7.1%, thin-slice computed tomography (CT) of the adrenal glands demonstrated no abnormalities.

Patient 3’s cortisol rhythm and ACTH levels were normal, except for hypercortisolism. Patient 3 had a history of diabetes.

**FIGURE 1:**
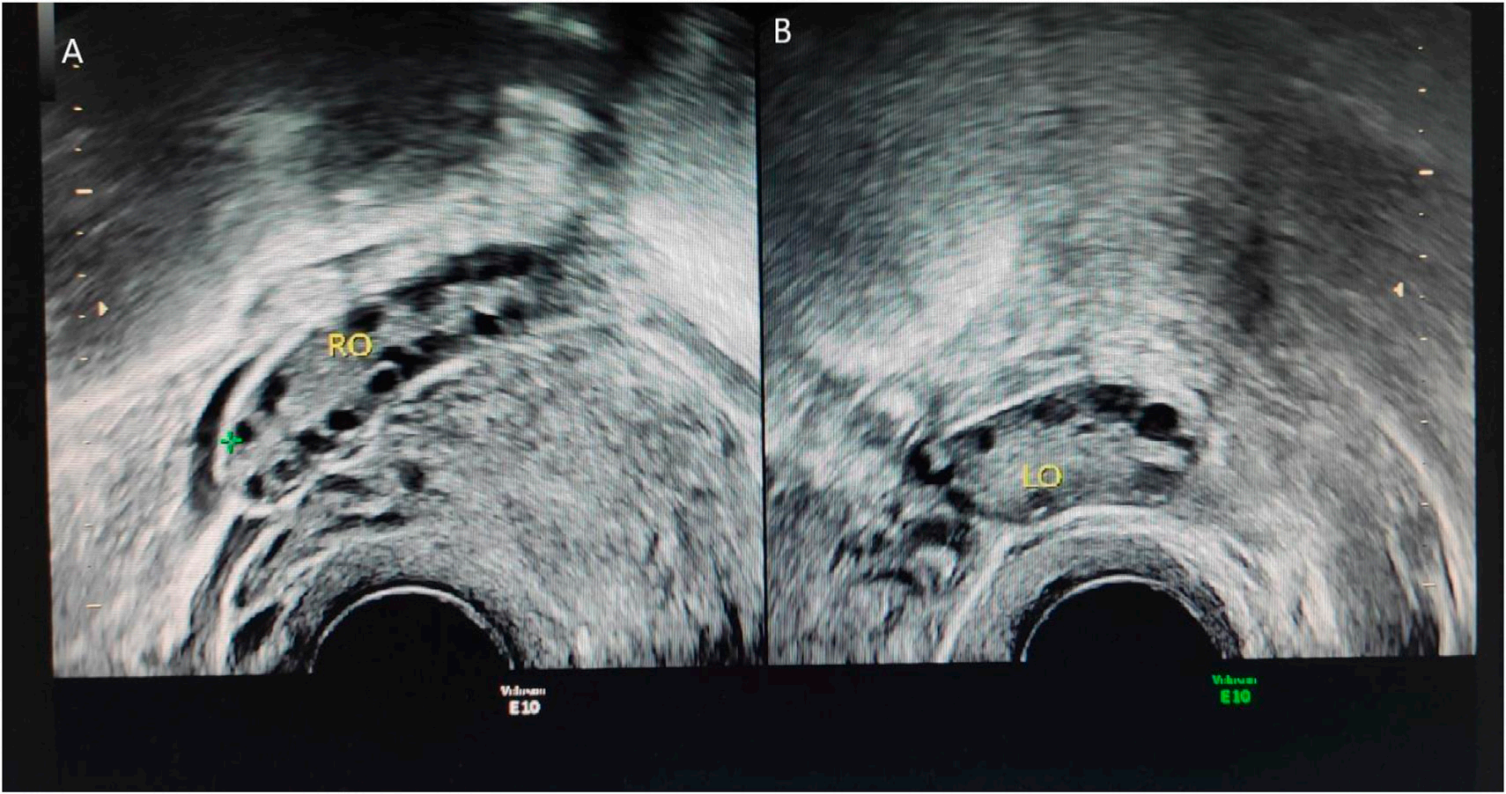
Ultrasound results of patient 4. Thin-slice CT of the adrenal glands demonstrates no abnormalities. **(A)** represents the right ovary of the patient. The right ovary is 22.4 × 9.9 mm, and approximately five follicles can be seen in the right ovary. The larger diameter is approximately 8.6 mm, and the smaller diameter is approximately 4.0 mm. **(B)** represents the left ovary of the patient, the size of the left ovary is 24.0 × 9.7 mm, and approximately four follicles can be seen in the left ovary, the larger diameter is approximately 7 mm, and the smaller diameter is approximately 4.6 mm indicates large volume of both ovaries accompanied by more follicles.

Patient 4: Gynecological color Doppler ultrasound: The uterine size was 42.7 × 41.0 × 34.8 mm. The left ovary was 35.0 × 16.0 mm; the right ovary was 36.5 × 16.6 mm, all demonstrated more than 12 follicles. Results: Several follicles were observed in ovaries. see Figure 1.

Data on insulin function in the four patients are depicted in Table 1, and data on related indicators of sex hormone parameters of the four patients are depicted in Table 2.

**TABLE 1:** Insulin function in the four patients (µIU/mL).

Category	0 min	30 min	60 min	120 min	180 min
Patient 1	3.14	26.17	32.11	37.93	42.66
Patient 2	11.0	169.13	349.54	113.82	6.98
Patient 3	3.231	23.15	40.65	59.27	68.89
Patient 4	19.72	147.3	>200	>200	>200

Note: Based on insulin release, all four patients had insulin resistance.

**TABLE 2:** Related indicators of the sex hormone parameters of the four patients.

Items	Patient 1	Patient 2	Patient 3	Patient 4	Clinical
Prolactin (PRL)	108	468.4	66.7	6.37	Follicular stage 66–490 Ovulation period 66–490 Luteal phase 66–490 Menopause 62–410
Follicle oietin (FSH)	5.71	4.286	0.167	11.09	Follicular stage 3.2–15 Ovulation period 7.5–20 Luteal phase 1.3–11 Menopause 36–138
Luteinizing hormone (LH)	5.98	4.764	<0.1	0.77	Follicular stage 1.2–12.5 Ovulation period 12–82 Luteal phase 0.4–19 Menopause 14–48
Progesterone	1.03	0.454	1.122	3.27	Follicular stage 0.4–2.3 Luteal phase 1.2–18.8 Menopause 0–1.4
Testosterone (TEST)	2,67	1.055	1.228	3.5	Follicular stage 0–1.0 Ovulation period 0–1.0 Luteal phase 0–1.0 Menopause 0–1.0

Note: All four patients had normal prolactin. Unit (mIU/mL). These four patients had normal FSH, LH, and progesterone. The patient 1, 2, and 3 had normal androgen and the fourth patient had elevated androgen.

These four patients had normal FSH, LH, and progesterone.

The patient 1, 2, and 3 had normal androgen and the fourth patient had elevated androgen.

### Gynecological ultrasound (ovulation)

**FIGURE 2:**
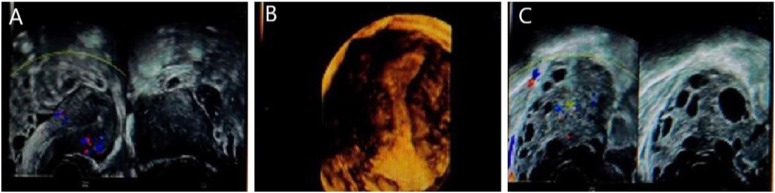
Gynecological ultrasound of patient 1. **(A)** represents the right ovary of the patient, with several echoless are as visible, approximately 19.5 × 19.0 mm. **(B)** represents uterus of the patient, and **(C)** represents more than 12 follicles in the left ovary, all less than 8 mm in diameter. Indicates large volume of the right ovary with multiple follicle-like echoes. More follicles in the left ovary, accompanied by pelvic effusion.

More than 12 follicles were observed in the left ovary, all of which were less than 8 mm in diameter, and several anechoic follicles were observed in the right ovary, which were approximately 19.5 × 19.0 mm in diameter. Results: The right ovary was large, with multiple follicular echoes. Several follicles were observed in the left ovary. Pelvic fluid was noted see Figure 2.

### QC of raw data

In the raw whole-exome sequencing data, the four patients had an average of approximately 70 M reads and the Q30 average was greater than 90%. Post quality control, an average of approximately 68 M high-quality reads was obtained, and the Q30 average was greater than 93%. The quality of the sequencing reads was high, and the high-quality sequencing data satisfied the downstream analysis. Summaries of high-quality sequences are presented in Supplementary Table S1; Supplementary Figure S1.

### Alignment to reference genome

High-quality sequence reads were aligned with the reference genome (hg19). Approximately 99% of the sequence reads obtained from the four patients aligned to the reference genome. Approximately 10% of duplicate reads were found in four patients. The whole exome region was sequenced with an average coverage of 99% for all four patients. The average coverage depth of the four patients was 110X, and the average 50X coverage region was 79%. The comparison was normal and met the requirements for identifying mutations. Quality control of the alignment of the four samples is depicted in Supplementary Table S2.

### Mutation identification

Genome Analysis Toolkit (GATK) was used to identify mutations in the four patients. On an average, 83,566 single-nucleotide polymorphisms (SNPs) were identified in each patient. Approximately 98% of the SNPs were annotated to the NCBI dbSNP database, and approximately 98% were annotated to the Genome Aggregation Database (gnomAD). The proportion of SNPs in exons was approximately 27%, and more than half of the SNPs were in the intronic region. The number of SNPs and annotations of the four patients are depicted in Supplementary Table S3.

On average, 13,333 indels were identified for each patient; approximately 90% of indels could be annotated to the NCBI dbSNP database, and approximately 92% of indels could be annotated to the gnomAD database. The proportion of indels in the exons was approximately 5%, and more than 78% of the indels were in the intronic region. The number of SNPs and annotations for the four patients are depicted in Supplementary Table S4. On average, 52 CNVs were identified in each patient, approximately 90% of which were located in the exonic region. Additionally, none of the CNVs were annotated in the CNVD database. The number of CNVs and annotations for the four patients are depicted in Table 3.

**TABLE 3:** Overview of the CNVs identified for four patients.

Type	Patient 1	Patient 2	Patient 3	Patient 4
downstream	1	1	1	1
exonic	53	37	43	63
intergenic		1		4
ncRNA_exonic	2	1	2	1
total	56	40	46	69
in CNVD database	0	0	0	0

### Identification of candidate causative genes and location

Mutations were filtered based on the mutation frequency in the population database. Considering the distribution of the four patients, we enumerated the intersections of gene mutations across the four patients. Three genes (mucin 4 (*MUC4*), FSHD region gene 1 (*FRG1*), and *AR*) were mutated in four patients. We found that *FRG1* had multiple mutations, and four missense mutations were annotated in the NCBI dbSNP database. *MUC4* and *AR* harbored indel mutations. Based on a literature search, we believe that *AR* (*AR*:NM_000044:exon1:c.173_174insGCAGCA:p. Q58delinsQQQ) is likely to be a genetic variant that could serve as a potential therapeutic target. To the best of our knowledge, this variant has not been reported previously. An overview of these three gene mutations is presented in Table 4.

**TABLE 4:** Overview of the six mutations identified in three genes for four patients.

Category	chr3	chr4	chr4	chr4	chr4	chrX
Start	195,510,749	190,883,039	190,883,075	190,883,080	190,883,081	66,765,161
End	195,510,749	190,883,039	190,883,075	190,883,080	190,883,081	66,765,161
Ref	—	T	C	C	T	—
Alt	GGAAGTGTCGGTGACATG AAGAGGGGTGGCATGAC CTGTGGATACTGA	C	T	A	G	GCAGCA
Gene.refGene	MUC4	FRG1	FRG1	FRG1	FRG1	AR
InterVar_automated		Uncertain significance	Uncertain significance	Uncertain significance	Uncertain significance	
ACMG (missense only)		PM2		PP3	PP3	
Patients detected	4	4	4	4	4	4
avsnp150		rs200007941	rs199625825	rs777434347	rs753433043	

### First-generation sequencing verification of the AR mutation sites

First-generation sequencing verification of the *AR* indel mutation.

**FIGURE 3:**
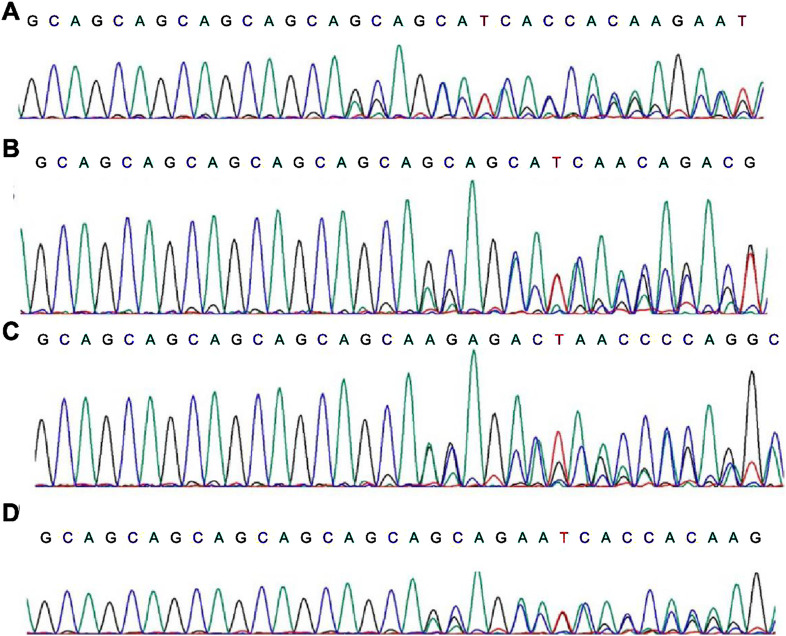
First-generation sequencing results of the *AR* mutation sites. **(A–D)** stands for patient 1, 2, 3, 4

Through first-generation sequencing (Sanger sequencing), we verified the *AR* indel mutations in these four patients (Figure 3).

## Discussion

PCOS is a common reproductive, endocrine, and metabolic disease with high genetic heterogeneity. The clinical manifestations can be accompanied by metabolic disorders, including glucose metabolism, obesity, and insulin resistance (Bell et al., 2021). Among all clinical manifestations, most PCOS patients have varying degrees of hyperandrogenemia. The high androgen level of the ovarian tissue in patients with polycystic ovaries can lead to infertility. Currently, PCOS incidence is increasing annually; however, the disease etiology and influencing factors have not been completely elucidated. This lack of understanding has increased the difficulty of clinical research and slowed progress in clinical diagnosis and treatment strategies. Therefore, it is vital to conduct further genetic studies.

Herein, four PCOS patients had abnormal blood sugar levels. Post treatment, such as hypoglycemia and weight loss, the patient’s symptoms significantly improved, and the same gene mutation was found. Patient 1 was administered oral metformin 0.25 g thrice a day, fasting blood glucose fluctuated between 5–6 mmol/L, and blood glucose fluctuated between 7–8 mmol/L 2 h post meals, and menstruation returned to normal. Patient 2 was administered oral metformin (0.5 g) thrice a day, acarbose 50 mg, thrice a day, fasting blood glucose fluctuated between 5–6 mmol/L, and blood glucose fluctuated between 7–8 mmol/L 2 h post meals, and menstruation returned to normal. Patient 3 had slightly higher blood sugar levels. After guiding diet and exercise, blood sugar levels and menstruation returned to normal. Patient 4: After a subcutaneous injection of a GLP-1 receptor agonist, the blood glucose and body weight of the patient reached standard levels, and menstruation was normal.

The detection of *AR* gene mutations in diabetic patients with PCOS indicates that mutations in the *AR* gene can lead to not only IR (Gao et al., 2020; Luo et al., 2021; Yu et al., 2020; Echiburú et al., 2020) but also PCOS. The influence of CAG repeat mutations of impacting testosterone *AR* gene on insulin resistance in women with PCOS has been previously reported (Lin et al., 2008). This offers a basis for early PCOS prevention and treatment.

The *AR* gene comprises 11 exons located at q11-q12 on the X chromosome. Exon 1 of the *AR* gene contains a polymorphic CAG repeat sequence, the number of which is typically between 10 and 35, which encodes the polyglutamine fragment of the *AR* transactivation domain. There is evidence that the CAG number is negatively correlated with *AR* transcriptional activity, and decreased *AR* sensitivity is associated with increased mortality and decreased blood glucose levels in patients with type 2 diabetes (Yu et al., 2014).

The expression of *AR* in PCOS is different in PCOS patients. The correlation between *AR* expression and serum FSH LH may be related to the number of follicles in PCOS (Lin et al., 2005). Previous studies have demonstrated that serine/arginine-rich splicing factors (SRSFs) mediate the role of *AR* in ovarian granulosa cells in patients with polycystic ovaries and it has been demonstrated that SRSFs are regulated by the corresponding microRNAs (miRNAs) (Rana et al., 2011). Alteration of SRSF expression interferes with the alternative splicing process of *AR*, which ultimately leads to a decline in *AR* function and ovarian androgens accumulation. Previous studies have demonstrated that *AR*s in PCOS patients is closely associated with hyperandrogenemia and abnormal follicular development. This study demonstrated that *CK2A* plays a role in PCOS development by phosphorylating and stabilizing *AR*, increasing *AR* expression, and increasing ovulation- and androgen synthesis-related gene expression (Supplementary Figure S2).

Using targeted next-generation sequencing (NGS), researchers studied DNA methylation patterns in the promoter regions of several genes, including anti-Müllerian hormone (AMH) and *AR*, in children born to 24 women with PCOS (12 of whom received metformin during pregnancy) and 24 women without PCOS in early infancy (2–3 months) (Möhlig et al., 2006). Female children demonstrated differences between groups at one CpG site of LEPR, two CpG sites of LEP, one CpG site of ADIPOR2, and two CpG sites of *AR*, whereas male children demonstrated differences in five CpG sites of LEP, three CpG sites of AMH, and nine CpG sites of *AR*, indicating that intrauterine PCOS environment can influence the sex-dependent acquisition of DNA methylation of certain genes.

Women with polycystic ovary syndrome have higher rates of miscarriage, gestational diabetes mellitus, gestational hypertension, preeclampsia, and cesarean section. Given the high prevalence of PCOS, exploring new biomarkers, perfecting diagnostic criteria, and improving treatment methods are essential to improve the accuracy and effectiveness of interventions, which will have a positive impact on patients’ lives (Stener-Victorin et al., 2024; Salari et al., 2024; Bahri Khomami et al., 2024). PCOS is associated with diverse risk factors in adults, including insulin resistance, type 1 or 2 diabetes, gestational diabetes, and mental health disorders. The limitations of this study include the lack of access to the genotype information of parents of the patients. Because this study aimed to investigate PCOS in a specific population, we had access to a limited sample size, which may have affected the generalizability of this study. Future studies with larger sample sizes are warranted.

## Conclusion

An indel mutation was identified in the AR (AR:NM_000044:exon1:c.173_174insGCAGCA:p. Q58delinsQQQ) was detected simultaneously in four patients, which provides new potential pathogenic sites and therapeutic targets for PCOS Patients with diabetes.

## Data Availability

The datasets presented in this article are not readily available because restrictions outlined in Regulations on the Management of Human Genetic Resources of the People's Republic of China. Requests to access the datasets should be directed to Corresponding author.
