# Supplementary material for: Molecular variants of multiple genes were revealed by whole-exome sequencing in PCOS patients with diabetes
Source: Front Genet. 2025 May 23;16:1541946. doi: 10.3389/fgene.2025.1541946 (PMC12141311; doi:10.3389/fgene.2025.1541946)
Supplement: Supplementary file 1 [file Supplementaryfile1.docx]

**Supplementary materials**

Supplementary Table 1 Sequencing data quality overview of 4 patients

| Category | Patient 1 | Patient 2 | Patient 3 | Patient 4 |
| --- | --- | --- | --- | --- |
| Raw reads(M) | 75.11 | 75.36 | 63.53 | 65.25 |
| Raw bases(Mb) | 11266.3 | 11304.31 | 9530.23 | 9787.3 |
| Raw Q20-R1(%) | 96.93 | 97.02 | 96.6 | 96.95 |
| Raw Q30-R1(%) | 92.2 | 92.33 | 91.93 | 92.52 |
| Raw Q20-R2(%) | 95.4 | 95.46 | 95.95 | 95.5 |
| Raw Q30-R2(%) | 89.3 | 89.3 | 90.34 | 89.44 |
| Raw Q20-all(%) | 96.17 | 96.24 | 96.28 | 96.23 |
| Raw Q30-all(%) | 90.75 | 90.81 | 91.14 | 90.98 |
| Clean reads(M) | 72.64 | 73.09 | 61.68 | 63.13 |
| Clean bases(Mb) | 10108.03 | 10250.5 | 7949.94 | 8640.07 |
| Clean Q20-R1(%) | 97.94 | 97.96 | 98.06 | 98.17 |
| Clean Q30-R1(%) | 93.71 | 93.71 | 94.14 | 94.35 |
| Clean Q20-R2(%) | 97.24 | 97.1 | 97.62 | 97.18 |
| Clean Q30-R2(%) | 91.81 | 91.48 | 92.74 | 91.73 |
| Clean Q20-all(%) | 97.59 | 97.53 | 97.84 | 97.67 |
| Clean Q30-all(%) | 92.76 | 92.59 | 93.44 | 93.04 |

Supplementary Table 2 Comparison of the quality control of the 4 patients

| Category | Patient 1 | Patient 2 | Patient 3 | Patient 4 |
| --- | --- | --- | --- | --- |
| Clean reads(M) | 72.64 | 73.09 | 61.68 | 63.13 |
| Duplication rate(%) | 9.91 | 9.68 | 11.01 | 10.39 |
| Total mapped reads(M) | 72.12 | 72.62 | 61.03 | 62.66 |
| Total reads mapping rate(%) | 99.29 | 99.35 | 98.94 | 99.26 |
| Coverage rate(%) | 99.74 | 99.75 | 99.74 | 99.74 |
| Target mean depth | 125.51 | 128.55 | 97.32 | 107.34 |
| T 4X coverage rate(%) | 99.66 | 99.66 | 99.64 | 99.64 |
| T 10X coverage rate(%) | 99.47 | 99.47 | 99.32 | 99.4 |
| T 20X coverage rate(%) | 98.75 | 98.8 | 97.78 | 98.39 |
| T 30X coverage rate(%) | 97.07 | 97.28 | 93.91 | 95.84 |
| T 10%X coverage rate(%) | 99.32 | 99.33 | 99.32 | 99.35 |
| T 20%X coverage rate(%) | 97.9 | 98.03 | 97.78 | 98.04 |
| T 30%X coverage rate(%) | 94.63 | 94.72 | 93.91 | 94.65 |
| T 50%X coverage rate(%) | 79.74 | 79.63 | 78.79 | 80.43 |

Supplementary Table 3 SNP quantity and annotation overview of 4 patients

| Category | Patient 1 | Patient 2 | Patient 3 | Patient 4 |
| --- | --- | --- | --- | --- |
| Total SNPs | 85732 | 85987 | 76110 | 86436 |
| NCBI dbSNP (SNPs) | 84637(98.72%) | 84763(98.58%) | 74648(98.08%) | 85217(98.59%) |
| 1000g_EAS | 79487(92.72%) | 79678(92.66%) | 70248(92.30%) | 80370(92.98%) |
| ExAC_EAS | 45903(53.54%) | 45776(53.24%) | 45107(59.27%) | 45571(52.72%) |
| gnomAD_exome_EAS | 45998(53.65%) | 45873(53.35%) | 45189(59.37%) | 45683(52.85%) |
| gnomAD_genome_EAS | 84269(98.29%) | 84386(98.14%) | 74347(97.68%) | 84934(98.26%) |
| 1/1(Hom) | 49394(57.61%) | 49936(58.07%) | 44045(57.87%) | 50416(58.33%) |
| 0/1 (Het) | 36267(42.30%) | 35983(41.85%) | 31996(42.04%) | 35942(41.58%) |
| Exonic | 22842(26.64%) | 22832(26.55%) | 22796(29.95%) | 22724(26.29%) |
| Splicing | 252(0.29%) | 257(0.30%) | 248(0.33%) | 263(0.30%) |
| UTR3 | 3183(3.71%) | 3275(3.81%) | 2766(3.63%) | 3230(3.74%) |
| UTR5 | 2263(2.64%) | 2268(2.64%) | 2070(2.72%) | 2283(2.64%) |
| Intronic | 50852(59.32%) | 51021(59.34%) | 42874(56.33%) | 51567(59.66%) |
| Intergenic | 2289(2.67%) | 2243(2.61%) | 1919(2.52%) | 2347(2.72%) |
| Upstream | 875(1.02%) | 878(1.02%) | 702(0.92%) | 895(1.04%) |
| Downstream | 412(0.48%) | 423(0.49%) | 326(0.43%) | 404(0.47%) |
| ncRNA_exonic | 784(0.91%) | 772(0.90%) | 702(0.92%) | 745(0.86%) |
| ncRNA_splicing | 3(0.00%) | 5(0.01%) | 3(0.00%) | 4(0.00%) |
| ncRNA_intronic | 1917(2.24%) | 1940(2.26%) | 1648(2.17%) | 1912(2.21%) |
| Synonymous SNVs | 11515(13.43%) | 11526(13.40%) | 11508(15.12%) | 11500(13.30%) |
| Nonsynonymous SNVs | 10746(12.53%) | 10682(12.42%) | 10656(14.00%) | 10599(12.26%) |
| Stopgain | 93(0.11%) | 95(0.11%) | 80(0.11%) | 94(0.11%) |
| Stoploss | 7(0.01%) | 12(0.01%) | 9(0.01%) | 9(0.01%) |
| Unknown | 492(0.57%) | 534(0.62%) | 555(0.73%) | 534(0.62%) |

Supplementary Table 4 Indels quantity and annotation overview of 4 patients

| Category | Patient 1 | Patient 2 | Patient 3 | Patient 4 |
| --- | --- | --- | --- | --- |
| Total indels | 14041 | 14002 | 11469 | 13822 |
| dbSNPs | 12744(90.76%) | 12631(90.21%) | 10246(89.34%) | 12473(90.24%) |
| 1000g_EAS | 8611(61.33%) | 8607(61.47%) | 6990(60.95%) | 8426(60.96%) |
| ExAC_EAS | 5497(39.15%) | 5416(38.68%) | 5229(45.59%) | 5427(39.26%) |
| gnomAD_exome_EAS | 5208(37.09%) | 5157(36.83%) | 4980(43.42%) | 5180(37.48%) |
| gnomAD_genome_EAS | 12972(92.39%) | 12889(92.05%) | 10414(90.80%) | 12728(92.09%) |
| 0/1 (Het) | 4784(34.07%) | 4760(34.00%) | 4031(35.15%) | 4753(34.39%) |
| Exonic | 694(4.94%) | 716(5.11%) | 682(5.95%) | 712(5.15%) |
| Splicing | 194(1.38%) | 187(1.34%) | 191(1.67%) | 195(1.41%) |
| UTR3 | 697(4.96%) | 695(4.96%) | 547(4.77%) | 705(5.10%) |
| UTR5 | 382(2.72%) | 386(2.76%) | 348(3.03%) | 384(2.78%) |
| Intronic | 10956(78.03%) | 10942(78.15%) | 8825(76.95%) | 10727(77.61%) |
| Intergenic | 299(2.13%) | 300(2.14%) | 235(2.05%) | 292(2.11%) |
| Upstream | 172(1.22%) | 164(1.17%) | 139(1.21%) | 193(1.40%) |
| Downstream | 83(0.59%) | 65(0.46%) | 47(0.41%) | 71(0.51%) |
| ncRNA_exonic | 108(0.77%) | 94(0.67%) | 76(0.66%) | 113(0.82%) |
| ncRNA_splicing | 1(0.01%) | 1(0.01%) | 0(0.00%) | 1(0.01%) |
| ncRNA_intronic | 403(2.87%) | 401(2.86%) | 328(2.86%) | 377(2.73%) |
| Frameshift insertion | 103(0.73%) | 100(0.71%) | 93(0.81%) | 93(0.67%) |
| Frameshift deletion | 127(0.90%) | 136(0.97%) | 120(1.05%) | 127(0.92%) |
| Nonframeshift insertion | 185(1.32%) | 206(1.47%) | 195(1.70%) | 195(1.41%) |
| Nonframeshift deletion | 211(1.50%) | 205(1.46%) | 206(1.80%) | 221(1.60%) |
| Stopgain | 8(0.06%) | 6(0.04%) | 4(0.03%) | 5(0.04%) |
| Stoploss | 1(0.01%) | 1(0.01%) | 1(0.01%) | 2(0.01%) |
| Unknown | 99(0.71%) | 102(0.73%) | 104(0.91%) | 110(0.80%) |


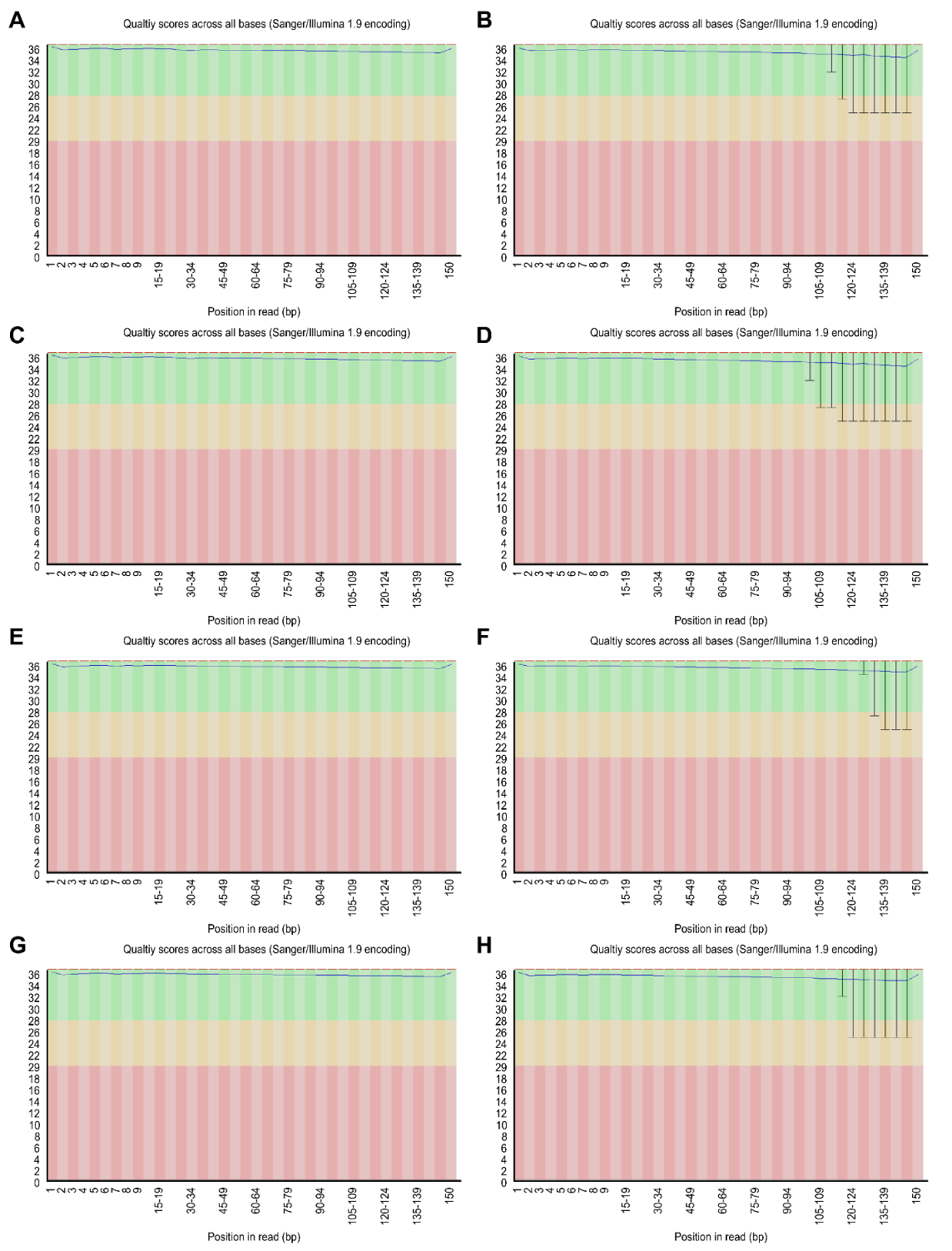


**Figure S1. Sequencing quality scores for raw sequence read data of whole-exome sequencing.** A-B: are the sequence quality distribution maps of reads 1 and 2 of patient 1; C-D: are the sequence quality distribution maps of reads 1 and 2 of patient 2; E-F: are the sequence quality distribution maps of reads 1 and 2 of patient 3; G-H: are the sequence quality distribution maps of reads 1 and 2 of patient 4.


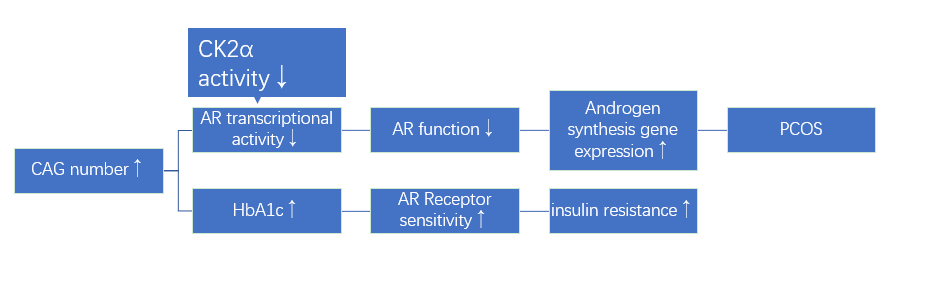


**Figure S2**. The role of *AR* gene in PCOS and insulin resistance
